# Ras inhibition by FTS attenuates brain tumor growth in mice directly and by enhancing reactivity of cytotoxic lymphocytes

**DOI:** 10.18632/oncotarget.420

**Published:** 2012-02-07

**Authors:** Elizabeta Aizman, Adi Mor, Ayelet Levy, Jacob George, Yoel Kloog

**Affiliations:** ^1^ Department of Neurobiology, The George S. Wise Faculty of Life Sciences, Tel Aviv University, 69978 Tel Aviv; ^2^ Department of Cardiology, Kaplan Medical Center, Rehovot, Israel

**Keywords:** Ras, Glioblastoma, CTL, FTS, Salirasib

## Abstract

One of the concerns in targeted drug therapy is that the inhibition of receptors and signaling molecules in tumor cells may also affect similar components in the tumor microenvironment or in the immune system, with undefined consequences for inhibition of tumor growth. Thus, in addition to its antitumor activity in mice and humans, the Ras inhibitor salirasib (S-farnesylthiosalicylic acid, FTS) also exhibits anti-inflammatory activity. Here we show three antitumor effects of FTS in immune-competent mice with subcutaneous or intracranial tumors. First, FTS exhibited antitumor activity in immune-competent, intracranial tumor-bearing mice and increased their survival relative to tumor-bearing immune-compromised mice. Second, FTS induced an increase in regulatory T cells in mouse splenocytes, but the inhibitory effects of FTS on tumor growth were not affected by these Foxp3+ T lymphocytes. Third, FTS increased antitumor T-cell reactivity by downregulating Foxp3. This caused TGF-β-dependent sensitization of the tumor to the immune system.

## INTRODUCTION

Foxp3, a transcription factor mainly expressed in CD25^+^CD4^+^ regulatory T cells (Tregs) [1], plays a major role in maintaining homeostasis in immune regulation by inhibiting the proliferation of effector T cells, thereby maintaining tolerance and preventing development of autoimmune diseases [2]. Recently we showed that expression of Foxp3 in T lymphocytes is negatively controlled by Ras [3-5]. Accordingly, Ras inhibition in lymphocytes, both *in vitro* and *in vivo*, induces an increase in Foxp3 expression in T cells [3-4]. In line with these findings, the Ras inhibitor salirasib (FTS) increases the number and function of Foxp3^+^ Tregs and consequently attenuates the progression of autoimmune diseases in experimental autoimmune encephalitis, an animal model for multiple sclerosis [6-7], and in type 1 diabetes [4, 8]. These results supported earlier findings showing effects of Ras inhibitors in autoimkmune diseases, including experimental autoimmune neuritis (an animal model for Guillain-Barré), the MRL/lpr mouse model for lupus, and experimental antiphospholipid syndrome in mice [9-12]. Notably, FTS inhibited all isoforms of active Ras (H-, K-, and N-Ras) and attenuated Ras signaling and Ras-dependent cell and tumor growth in animal studies [13-14]. In recent clinical trials (phases I and II) in patients with pancreatic or nonsmall cell lung cancer, FTS exhibited marked efficacy with limited toxicity (http://www.concordiapharma.com). Although Foxp3 was thought to be unique marker for Tregs [2], it was found to be expressed also by nonlymphocytic nonhematopoietic cells and by cancer cells [15-16]. Foxp3 expression has been demonstrated in breast cancer cells, melanoma cells, virally transformed B cells, and in cells derived from a variety of solid tumors [15-18]. The effects of FTS-induced Ras inhibition on Foxp3 expression in tumor cells and its influence on their growth are not known. Our main objective in the present work was to examine these effects.

Although Foxp3^+^ Tregs have been found to have positive effects in autoimmune diseases (inhibition of T effector cells and attenuation of the disease), their accumulation in tumors is associated with unfavorable clinical prognosis [19]. Foxp3^+^ Tregs in tumors inhibit activation of the antitumor immune response. Moreover, depletion of Foxp3^+^ Tregs results in activation of CD8^+^ cytotoxic T lymphocytes (CTLs) and enhances their infiltration into tumors [19]. These effects are accompanied by complete regression of tumors [20-21]. Thus, the two major functional characteristics of FTS, inhibition of tumor growth and attenuation of autoimmunity, seem potentially to pose a therapeutic dilemma. On the one hand FTS inhibits cancer cell proliferation and tumor growth [23, 24]; on the other hand it upregulates Foxp3^+^ Tregs [3, 4, 5], thereby attenuating autoimmune disease but inhibiting the antitumor activity of CTLs [22].

Our working hypothesis in the present study was that the functions of Ras in autoimmune diseases differ from its functions in cancer. We postulate that in cancer FTS has a dual effect: it causes a unique Treg-mediated immune response, which has a favorable effect on tumor cells while at the same time attenuating tumor-cell growth. To test our hypothesis, we examined the effect of FTS on tumor growth in immune-compromised and immune-competent mice. According to our hypothesis, we expected to find stronger antitumor activity of FTS in the immune-competent mice owing to the presence of immune cells in these mice.

Glioblastoma (GBM) is one of the commonest and most aggressive neoplasms among human primary brain tumors [25-26]. Using the mouse glioma cell line GL261 [27], we examined the growth of these cells in syngeneic C57bl/6 immune-competent mice (host) and nude mice.

Our results showed that FTS treatment significantly inhibited tumor growth when these GBM cells were implanted either subcutaneously (s.c.) or intracranially into the immune-competent C57bl/6 mice. Thus, in mice with intracranial tumors, FTS not only decreased tumor size but also prolonged survival. In tumor-bearing nude mice, however, the life span of animals treated with FTS did not differ from those that remained untreated. In line with our hypothesis, FTS reduced the expression of Foxp3 in tumor cells. This reduction may have altered the tumor microenvironment by enhancing the antitumor immune response. These results point to the intriguing possibility of a mechanism in which Ras inhibition reduces resistance of tumors to immune-mediated protection. These novel findings also provide strong support for the treatment of human glioblastoma with FTS.

## RESULTS

### FTS inhibits proliferation of GL261 cells and decreases levels of K-Ras-GTP, P-Erk and P-Akt *in vitro*

GL261 cells are mouse glioma cells that carry point mutations in the *Kras* and *p53* genes [27]. These cells therefore seemed suitable for studies on the cross-talk between cancer cells and immune cells in an immune-competent syngeneic mouse model.

**Figure 1:**
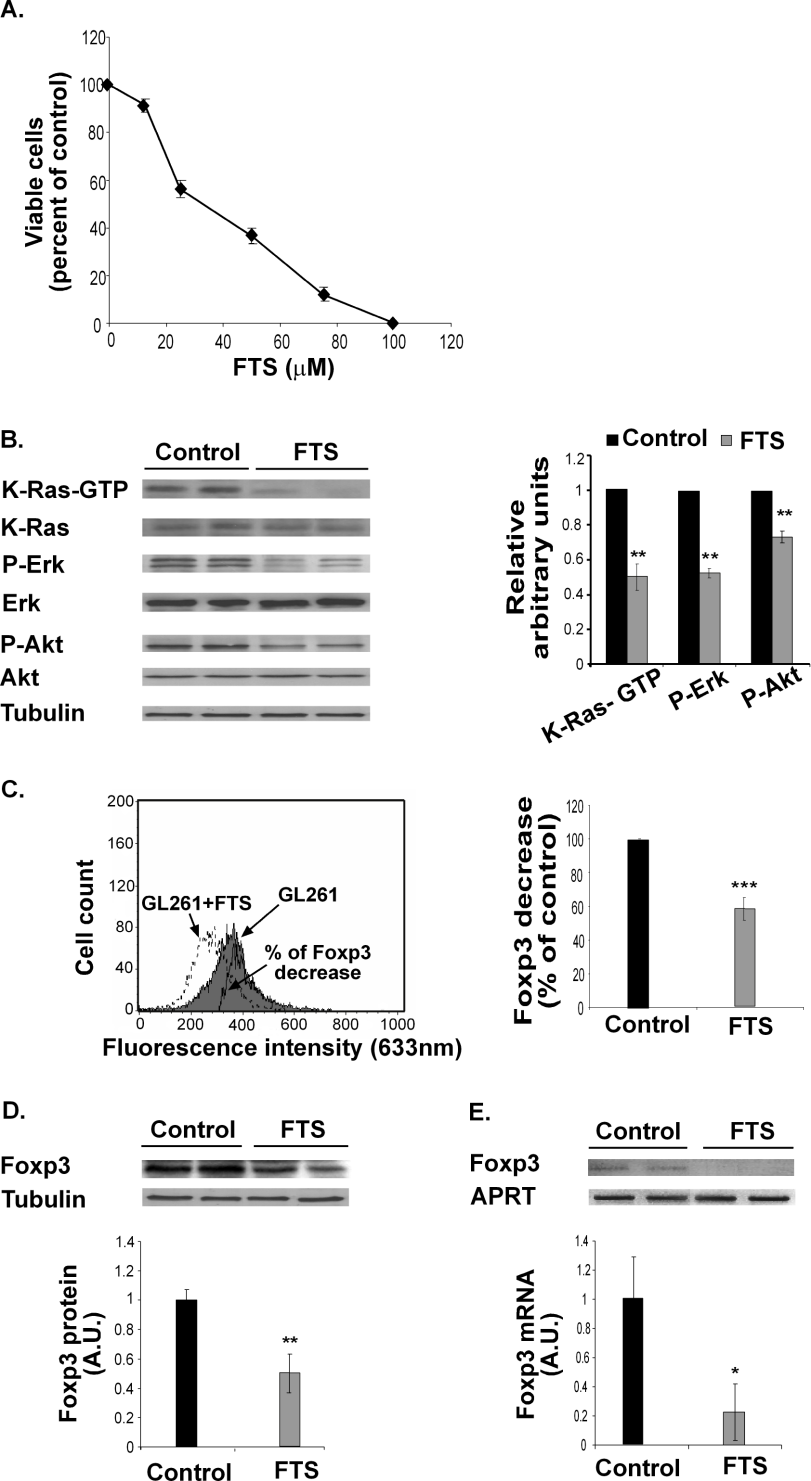
FTS inhibits GL261 cell proliferation and decreases K-Ras-GTP, P-Erk, P-Akt and Foxp3 *in vitro* (A) GL261 cells were untreated (control) or treated for 3 days with 12.5, 25, 50, or 100 μM FTS, and the cells were then directly counted. A typical inhibition curve is shown (means ± SEM, n=5). (B) GL261 cells were treated with FTS for 3 days and then assayed for K-Ras, K-Ras-GTP, Erk, P-Erk, Akt, P-Akt and tubulin by SDS-PAGE and western blotting, as described in Methods. Representative blots are shown (*left*). Densitometry for K-Ras-GTP, P-Erk, and P-Akt is presented (means ± SD; *right*). **, p<0.01 compared with control (n=7). (C) GL261 cells were treated with FTS or left untreated (control) for 24 hours and then assayed for Foxp3 by flow cytometry (*left*). Statistical analysis of the results is presented as means ± SD (*right*). ***, p<0.0001 compared with control (n=7). (D) FTS-treated GL261 cells were assayed for Foxp3 by western blotting (*top*). Statistical analysis of the results is presented as means ± SD (*bottom*). **, p<0.001 compared with control (n=7). (E) FTS-treated GL261 cells were assayed for Foxp3 and APRT mRNA by RT-PCR. Representative gels are shown (*top*). Densitometry for Foxp3 (means ± SD) is shown (*bottom*). *, p<0.05 compared with control (n=5). A.U., arbitrary units.

We first investigated the effect of FTS on GL261 cells *in vitro*. FTS (12.5–100 μM) inhibited GL261 proliferation in a dose-dependent manner (IC_50,_ 43.2±3.3 μM; 4 separate experiments, each performed in triplicate; Figure 1A). We then examined whether the reduction in cell proliferation was associated with downregulation of Ras and its major downstream signals. Western blot analysis of viable cells with specific Abs revealed that treatment with FTS (50 μM) decreased the levels of K-Ras-GTP, P-Erk, and P-Akt by 48.26%±7.5%, 46.9%±2.67%, and 37.82%±4.02%, respectively (Figure 1B).

### FTS decreases Foxp3 mRNA and protein expression in GL261 cells *in vitro*

Next we examined whether GL261 cells self-express Foxp3, and investigated the possible effect of FTS on any such expression. FACS analysis and western blot assays with anti-Foxp3 Ab revealed that GL261 cells express large amounts of Foxp3 (Figure 1C, D). Treatment with 50 μM FTS for 24 hours decreased Foxp3 levels by 52.3%±9% and 41.3%±5% as determined by FACS and by western blot analysis, respectively (Figure 1C, D). The same treatment also resulted in a marked decrease in Foxp3 mRNA (by 80%±18.3%; Figure 1E). These results indicated that FTS, as opposed to its effects in T lymphocytes [3-4, 8], induces downregulation of Foxp3 in GL261 glioma cells.

### FTS inhibits subcutaneous GL261 tumor growth in C57bl/6 mice

**Figure 2:**
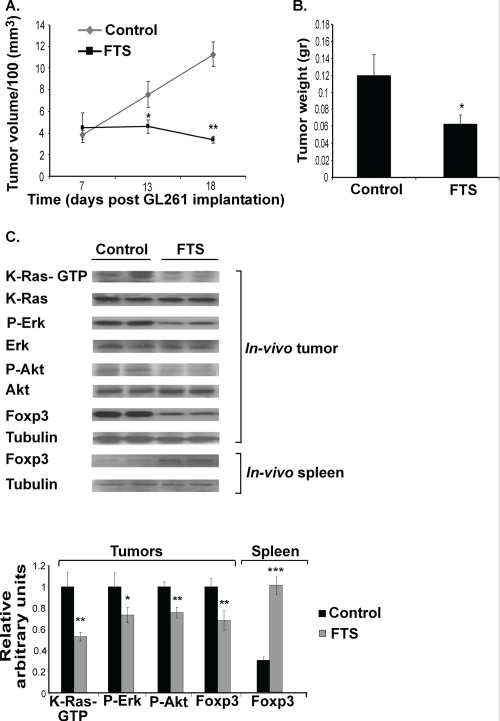
FTS inhibits the growth of subcutaneous GL261 tumors and decreases their downstream Ras proteins and their Foxp3 levels *in vivo* GL261 cells were implanted s.c. in the right flank of C57bl/6 mice, which were then treated for 12 days with oral FTS or vehicle. After the mice were killed the volumes and weights of their excised tumors were recorded, as described previously [31]. (A) Tumor volumes in FTS-treated and control mice are presented as means ± SEM. *, p<0.05, **, p<0.01 compared with control. (B) Tumor weights in FTS-treated and control mice are presented as means ± SEM. *, p<0.05 compared with control. (C) K-Ras, K-Ras-GTP, Erk, P-Erk, Akt, P-Akt, Foxp3 and tubulin in tumors, and Foxp3 and tubulin in splenocytes, of FTS-treated (n=10) and vehicle-treated mice (n=10) were assayed by immunoblotting. Representative blots are shown (*top panel*). Densitometry values for K-Ras-GTP, P-Erk, P-Akt, and Foxp3 are presented as means ± SEM (*bottom panel*). *, p<0.05, **, p<0.01, ***, p<0.001 compared with control.

Our next aim was to investigate the effect of Ras inhibition by FTS on GL261 tumor cells in a syngeneic mouse model with a competent immune system. C57bl/6 mice were implanted s.c. with 2×10^6^ GL261 cells (see Methods). After 7 days the mice were randomly divided into two groups (n=40 in each group) that were treated daily for 12 days with oral FTS (60 mg/kg) or vehicle, after which their excised tumors were weighed and examined by western blotting and FACS, as described in Methods. Figure 2A shows the changes in tumor volumes as a function of time. Compared with the control, tumor volume in the FTS-treated immune-competent mice was significantly inhibited (by 69%±5%; Figure 2A). Tumor weight in the FTS-treated mice was decreased by 47.8%±3.8% relative to controls (Figure 2B).

The *in-vivo* results of biochemical analyses of K-Ras-GTP, P-Erk, and P-Akt in the excised tumors were similar to those obtained *in vitro*, i.e., their levels were significantly decreased (by 47.06%±3.9%, 26.74%±7.05%, and 24.45%±5.03%, respectively; Figure 2C). Foxp3 levels in these glioma cells were also significantly decreased (by 31.88%). However, Foxp3 levels in the splenocytes of the same tumor-bearing FTS-treated mice were significantly increased (by 69.3%±8.63%; Figure 2C). This result is in agreement with recent findings that FTS upregulates peripheral Foxp3^+^ regulatory T cells [3-4, 8].

### Inhibition of GL261 tumor growth by FTS is not affected by FTS-induced Foxp3 Tregs

The antitumor response of CD8^+^ CTLs is weakened by an increase in CD25^+^Foxp3^+^ Tregs [19]. Seeing that FTS causes an increase in Foxp3 expression in lymphocytes [3-2, 8], we suspected that this might interfere with the antitumor effect of FTS. Thus, depletion of Tregs by the specific anti-CD25 Ab might induce an antitumor immune response and hence enhance the FTS-induced inhibition of tumor growth [19].

**Figure 3:**
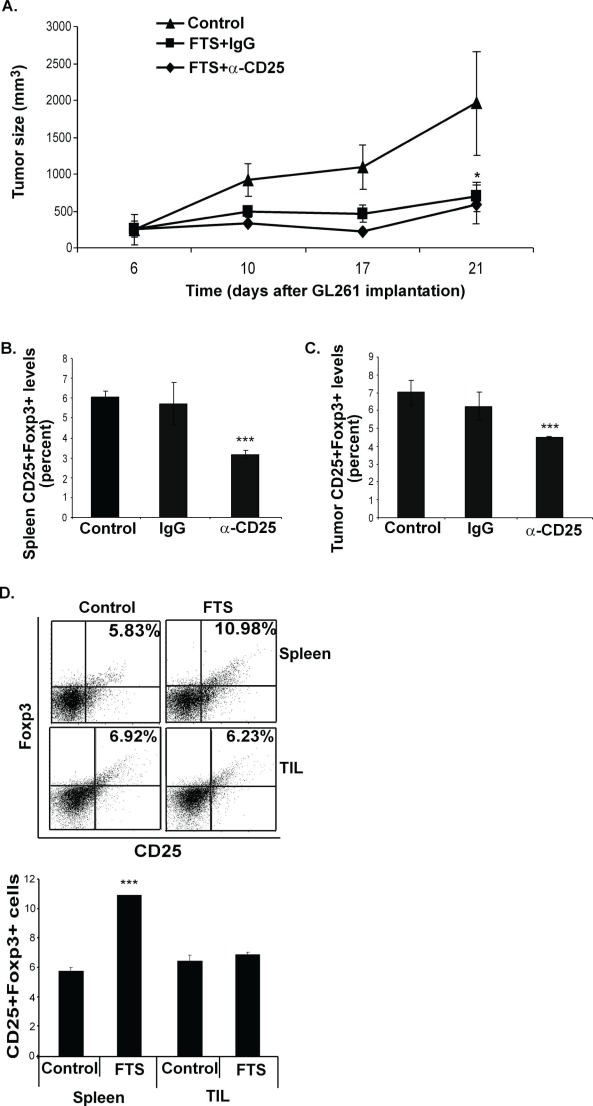
Inhibition of GL261 tumor growth by FTS is not affected by FTS-induced Foxp3 Tregs (A-C) C57bl/6 mice were implanted s.c. with GL261 cells, treated with FTS and anti-CD25 Ab, or FTS and anti-IgG, or vehicle only, and were killed on day 21, as described in Results. (A) Tumor volumes, measured during and at the end of treatment, are shown as means ± SEM. *, p<0.05 compared with control (untreated) mice. (B) CD25^+^Foxp3^+^ T cells in the spleen were assayed by flow cytometry. Statistical analysis of the results, presented as means ± SEM, shows that cell numbers in anti-CD25-treated mice were decreased by 44.55%±5.3% and by 47.54%±8.6% compared with anti-IgG1-treated and untreated control mice, respectively). ***, p<0.001 in both cases. (C) CD25^+^Foxp3^+^ T cells in tumors were assayed by flow cytometry. Statistical analysis of the results, presented as means ± SEM, shows that cell numbers in anti-CD25-treated mice were decreased by 28%±7.6% and by 36.03%±3% compared with anti-IgG1-treated and untreated control mice, respectively). ***, p<0.0001 in both cases. (D) C57bl/6 mice were implanted with GL261 cells and were then separated into two groups, treated daily with FTS or vehicle (control), and killed on day 21, as described in Results. Their excised spleens and GL261 tumors were assayed for CD25^+^Foxp3^+^ by flow cytometry (*upper panel*). Statistical analysis of the results is shown (*lower panel)*. ***, p<0.001 compared with control.

To examine this possibility we used C57bl/6 mice implanted s.c. with GL261 cells as described above. On day 4 after tumor cell implantation the mice were divided into three groups. To decrease the number of Tregs, mice in the first group (n=8) were injected i.p. with 250 μg of anti-CD25 Ab on days 4 and 11. At the same times, mice in the second group (control, n=8) received 250 μg of IgG1 Ab. On day 5, mice in those two groups each received 60 mg of oral FTS. The third group (n=8) served as a control for the FTS treatment and received vehicle only. Tumor volumes were determined on days 6, 10, 17 and 21, and the data are presented in Figure 3A. Tumor growth was inhibited in the two groups of FTS-treated mice, and by day 21 the decrease in tumor volume in both of these groups relative to the vehicle-treated control was highly significant (decrease of 70.25%±19% in the first (anti-CD25 Ab-treated) group (p<0.05) and 64.69%±15.6% (p<0.05) in the second (anti-IgG1 Ab-treated) group; Figure 3A). However, there was no difference in tumor volume between the two FTS-treated groups (Figure 3A), suggesting that the specific depletion of CD25^+^Foxp3^+^ Tregs did not enhance the antitumor activity of FTS. Importantly, in the anti-CD25 Ab-treated group we detected, as expected, a marked decrease in CD25^+^Foxp3^+^ Tregs both in the spleens (Figure 3B) and in the tumors (Figure 3C) of the treated mice. Thus, the FTS-induced presence of Tregs did not interfere with the antitumor activity of the drug. This conclusion was supported by the results of experiments in which CD25^+^Foxp3^+^ Tregs in the spleens and in the GL261 tumors of C57bl/6 mice were assayed without anti-CD25 Ab and with or without FTS treatment (Figure 3D).

In another experiment, C57bl/6 mice implanted s.c. with GL261 cells were treated daily, from 7 days after implantation, with FTS or vehicle. On day 18 after implantation the mice were killed and CD25^+^Foxp3^+^ levels in their excised spleens and tumor-infiltrating lymphocytes (TIL) were measured by flow cytometry (see Methods). In spleens from FTS-treated mice, the numbers of CD25^+^Foxp3^+^ regulatory T cells had increased (by 203.7%±9.3% relative to controls; Figure 3D), in agreement with our previously reported results [3-4, 8]. However, treatment with FTS had no effect on the numbers of CD25^+^Foxp3^+^ Tregs in the tumors (Figure 3D), indicating that Tregs did not migrate to the tumors or proliferate as a result of FTS treatment. Quantification of the results is shown in Figure 3D.

### FTS decreases secretion of the immunosuppressive cytokine TGF-β from GL261 cells

**Figure 4:**
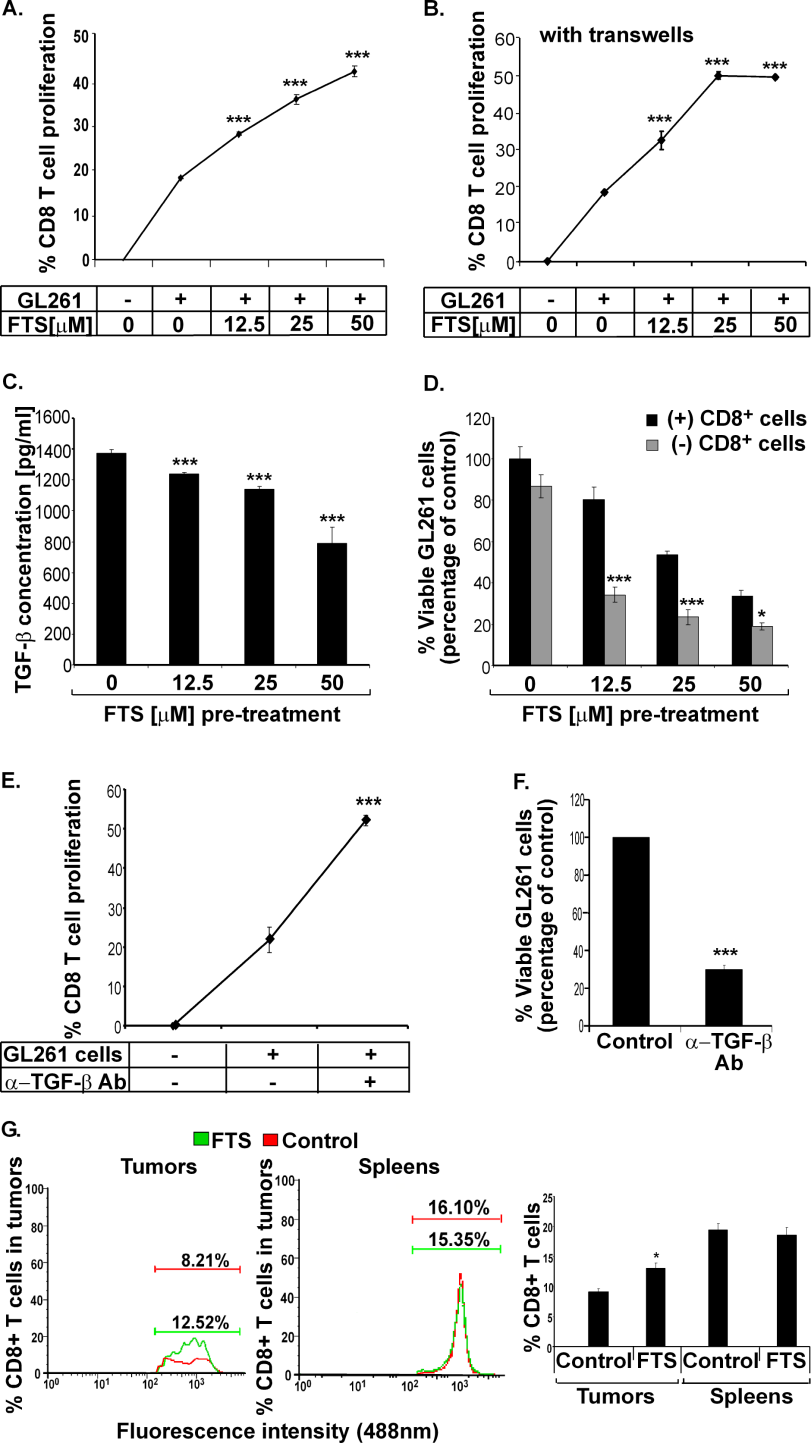
FTS decreases secretion of the immunosuppressive cytokine TGF-β from GL261 glioma cells and increases the proliferative and cytotoxic capacities of CTLs *in vitro* as well as accumulation of CTLs in the tumors *in vivo* (A) GL261 tumor cells were treated *in vitro* for 24 hours with FTS or CD8 or both, as described in Results. The cells were then washed thoroughly, and CFSE-labeled CD8^+^ T cells, (isolated from FTS- or vehicle-pretreated GL261 tumor-bearing mouse splenocytes) were added and cocultured with the FTS-pretreated GL261 cells for 96 hours. The rate of CTL proliferation was measured by flow cytometry. Statistical analysis of the results is presented as means ± SEM (n=8). ***, p<0.001 compared with vehicle-treated mice. (B) The experiment was performed as in *A*, except that the CD8^+^ T cells were now separated from the GL261 tumor cells by a transwell, preventing cell passage. The rate of CD8^+^ T cell proliferation was measured by flow cytometry. Statistical analysis of the results is presented as means ± SEM (n=8). ***, p<0.001 compared with vehicle-treated mice. (C) GL261 tumor cells were treated with the indicated doses of FTS or with vehicle (control) for 24 hours and then assayed for TGF-β (see Methods). The ELISA results are shown (means ± SEM, n=8). ***, p<0.001 compared with vehicle-treated control cells. (D) Isolated CD8^+^ T cells were cocultured with FTS-pretreated GL261 cells for 96 hours and their proliferation was analyzed, as described in Methods. Numbers of viable CTLs are presented as means ± SEM (n=8). *, p<0.05, ***, p<0.001 compared with vehicle-treated cells. (E-F) GL261 tumor cells were incubated with CFSE-labeled-CD8^+^ T cells, with or without TGF-β-blocking anti-TGF-β Ab, for 96 hours. The rate of CD8^+^ T cell proliferation was measured by flow cytometry (E), and viable GL261 cells were counted (F). Statistical analysis of the results is presented as means ± SEM (n=5). ***, p<0.001 compared with cells not treated with anti-TGF-β Ab. (G) C57bl/6 mice implanted s.c. with GL261 tumor cells were divided into two groups for treatment with FTS (n=10) or vehicle (n=10), as described in Methods. The mice were killed 21 days after the cells were implanted and their tumors and spleens were assayed for CD8^+^ T cells by flow cytometry (*left* and *middle*). Statistical analysis of the flow cytometry results (n=10) is presented (*right*). *, p<0.05 compared with vehicle-treated controls.

The lack of involvement of the immune system's regulatory arm in the antitumor activity of FTS (Figure 3), together with the finding that Foxp3 in GL261 glioma cells was decreased after FTS treatment, suggested that the Foxp3-depleted GL261 glioma cells might behave like lymphocytes, and accordingly produce a pro-inflammatory microenvironment. To pursue this idea, we studied the possible involvement of the immune system's inflammatory arm; more specifically, we wanted to find whether the FTS-induced downregulation of Foxp3 expression observed in GL261 cells (see Figure 2) has implications for the activity of CD8^+^ T cells. To examine this possibility, we first isolated CD8^+^ T cells from the spleens of GL261-tumor-bearing mice treated with FTS for 1 week, and labeled the isolated cells with the fluorescent dye carboxyfluorescein succinimidyl ester (CFSE; see Materials and Methods). We then added them to GL261 cells that had been pretreated for 24 h with vehicle or increasing doses of FTS and then thoroughly washed. Figure 4 shows that after 96 hours, CD8^+^ T cells incubated with the FTS-pretreated GL261 cells attained significantly higher proliferation rates than CD8^+^ T cells incubated with untreated control GL261 cells. The rate was dose dependent, yielding an increase in CD8^+^ T cells proliferation of 153.3%±1.55%, 184.8%±6.3%, and 228.8%±6.6% in the presence of 12.5, 25 and 50 μM FTS, respectively (Figure 4A). Similar results were obtained when we used transwells to separate the CD8^+^ T cells from the GL261 cells and treated the cells with the same FTS dosages as above. In this case, the proliferation rates of CD8^+^ T cells were increased by 175.6%±13.51%, 270.2%±5.4%, and 267.56%±2.7%, respectively, relative to controls (Figure 4B). These results suggested that FTS reduces an anti-inflammatory response in the GL261 cells, and that this FTS effect might be mediated by a small soluble molecule that is secreted into the media and diffuses through the transwells.

Our next task, therefore, was to identify this putative soluble anti-inflammatory molecule. We considered that a possible candidate might be transforming growth factor (TGF)-β, well known as an anti-inflammatory cytokine with a pivotal role in the growth and progression of gliomas [28]. Notably, glioma cells are known to induce immune suppression via the production of interleukin-10 (IL-10) and TGF-β [29]. We recently showed, moreover, that FTS directly perturbs TGF-β signaling to Smad-dependent and Erk-dependent pathways in neurofibromin-deficient cells [30]. We therefore examined the effect of FTS on the secretion of TGF-β from GL261 cells (see Methods). We found that FTS induced a dose-dependent decrease in TGF-β secretion from GL261 cells (by 9.82%±0.76%, 17.52%±1.27%, and 42.67%±7.63% in the presence of 12.5, 25 and 50 M FTS, respectively;, Figure 4C). Accordingly, we postulated that the increase in proliferation of CD8^+^ T cells observed after their incubation with FTS-pretreated GL261 cells (Figure 4A, B) reflects a decrease in TGF-β secretion.

To find out whether the abovementioned CD8^+^ T cells contribute to the growth-inhibitory effect of FTS on the GL261 cells, we cocultured the CD8^+^ T cells (isolated, as described above, from FTS-pretreated GL261 tumor-bearing mice) with FTS-pretreated GL261 cells for 96 hours, then removed the CD8^+^ T cells and analyzed the viability of the GL261 cells (see Materials and Methods). The FTS-pretreated GL261 cells that were co-cultured with CD8^+^ T cells exhibited significantly lower viability than GL261 cells that were not incubated with CD8^+^ T cells (Figure 4D). The IC_50_ of FTS-pretreated GL261 cells that were incubated with CD8^+^ cells was significantly lower than that of the nonincubated FTS-pretreated GL261 cells (14.27±1.3 μM vs. 34.87±3.4 μM FTS, Figure 4D). Taken together, these results demonstrated that growth inhibition by FTS enhances the cytotoxicity of CD8^+^ T cells.

To support the apparent connection between the enhanced proliferative and cytotoxic capacities of CD8^+^ T cells and the presence of the TGF-β cytokine, we examined the proliferative and the cytotoxic effects of CD8^+^ T cells with and without neutralization of the TGF-β expression from GL261 cells. TGF-β was neutralized as described in Methods. The results (Figure 4E, F) show that neutralization of the TGF-β expressed by GL261 cells indeed significantly increased the proliferation of CD8^+^ T cells (by 175.23%±33.62, Figure 4E) and decreased their cytotoxic activity (a decrease of 79.5%±4.65, Figure 4F). The neutralization percentage of TGF-β in GL261 cells was 72% (data not shown).

### FTS increases the accumulation of CD8^+^ CTLs within GL261 tumors

Having established that FTS enhances both the proliferative and the cytotoxic effects of CD8^+^ CTLs *in vitro*, we wanted to find out whether FTS had the same effect on these T cells *in vivo*. We examined whether FTS affects the accumulation of CD8^+^ T cells in peripheral GL261 tumors and in the spleens of FTS-treated mice. Our results show that FTS significantly increased the numbers of CD8^+^ CTLs in the tumors (by 144.9%±7.78%, p<0.05; Figure 4G), identifying FTS as a positive regulator of the migration and accumulation of CD8^+^ CTLs in the tumors. However, no significant differences between CD8^+^ T cell numbers were observed in the spleens of FTS- and vehicle-treated (control) mice (Figure 4G). Thus, unlike the effect of FTS in enhancing the splenic production of Tregs, FTS had no such effect on the splenic production of CTLs.

### FTS prolongs survival of intracranial immune-competent tumor-bearing mice but not of tumor-bearing nude mice

**Figure 5:**
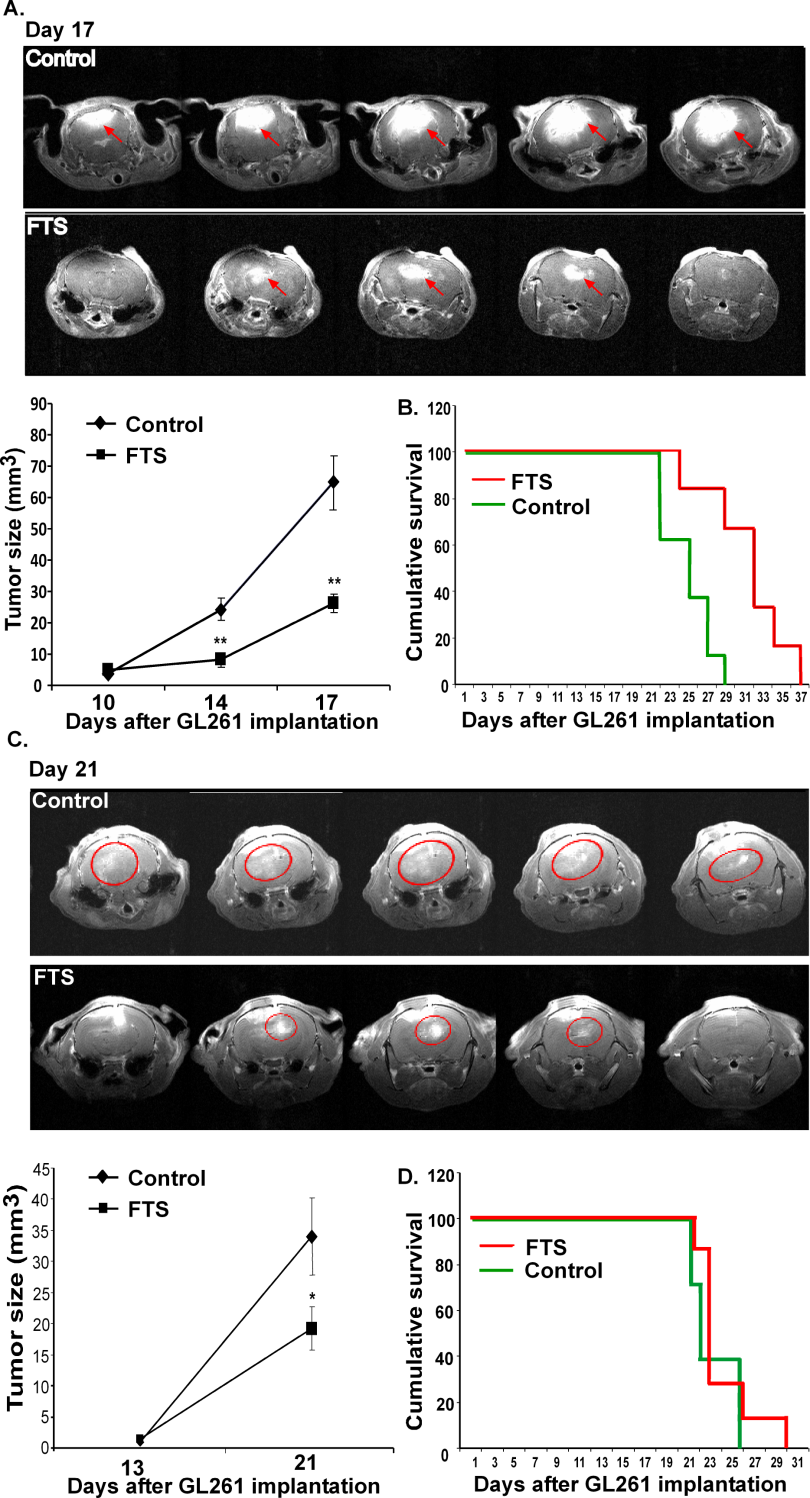
FTS inhibits intracranial tumor growth and prolongs survival of intracranial immune-competent tumor-bearing mice but not of the tumor-bearing nude mice GL261 cells were implanted into the crania of C57bl/6 immune-competent and nude mice. Mice were treated and their survival was assessed as described in Results. Gadolinium-enhanced regions were defined and their volume (in mm^3^) and were accumulated. (A) Representative images of brains of immune-competent and nude mice are presented (*top panel*) (n=8 in each group). Statistical analysis of the results is presented as means ± SEM (*bottom, left*). * p<0.05, ** p<0.01 compared with vehicle-treated controls. (B) Survival of the intracranial tumor-bearing immune-competent mice (n=8 in each group) was monitored and Kaplan-Meier survival curves are presented; p=0.007, log-rank significance test. (C) Representative images of brains of nude mice are presented (*top panel*) (n=8 in each group). Statistical analysis of the results is presented as means ± SEM (*bottom, left*). ** p<0.01 compared with vehicle-treated controls. (D) Survival of the intracranial tumor-bearing nude mice (n=8 in each group) was monitored and Kaplan-Meier survival curves are presented; p=0.415, log-rank significance test.

As mentioned earlier, GL261 cells were chosen for the present experiments to examine the effects of FTS with and without immune system involvement. Previous studies have shown that FTS is able to inhibit intracranial U87 glioblastoma cell growth in nude mice but not to prolong their survival [31]. In the present study we compared the effect of FTS on GL261 glioma intracranial tumors in immune-competent (C57bl/6 mice) with its effect in immune-compromised mice (nude mice). GL261 cells were implanted into the crania of nude mice and of C57bl/6 mice (syngeneic hosts), as described in Methods. Four days later we divided the mice randomly into two groups (n=8 per group). Mice in one group were treated with oral FTS (60 mg/kg) and the other (control) with the carboxymethyl cellulose vehicle. Tumor growth was assessed by gadolinium-DTPA-enhanced T1-weighted MRI (see Materials and Methods) on days 10, 14 and 17 in C57bl/6 mice and on days 13 and 21 in nude mice. Typical images presented in Figure 5A (immune-competent mice) and Figure 5C (nude mice) indicate that tumor growth was attenuated in both models. However, FTS exerted a significantly stronger inhibitory effect in the immune-competent C57bl/6 mice than in the nude mice. In C57bl/6 mice the tumor volume was decreased by 66.6%±3.44% and 59.67%±15.89%, respectively, on days 14 and 17 (p<0.01 on both days, Figure 5A). The decrease in the nude mice was smaller (only 43.57%±19.58% on day 21; p<0.05, Figure 5C).

It is important to note that FTS treatment increased survival rates only in the immune-competent C57bl/6 mice (p=0.007, log-rank significance test; Figure 5B). Kaplan-Meier survival curves obtained in these experiments are presented in Figure 5B (C57bl/6 mice) and Figure 5D (nude mice).

## DISCUSSION

The concept of selective targeting of signal transduction pathways has been developed over the past few years and has proved highly successful. Thus, anti-Bcr Abl drugs (e.g. imatinib), anti-epidermal growth factor receptor drugs (e.g. Erbitux and Tarceva®), anti-ERB2 drugs (e.g. trastuzumab) or anti-VEGF receptor drugs (e.g. bevacizumab, semaxanib) have been developed and approved for a variety of cancer types [32, 33-36]. Other drugs, not only against receptors but also against their downstream targets, are now being developed. These drugs include the Ras inhibitor salirasib (FTS), for which clinical phase I and II clinical trials were recently completed in patients with pancreatic or NSCLC. Ras oncogene products that are targeted by FTS are involved in more than 30% of all human tumors [37]. They are therefore considered to be good targets for cancer therapy [38].

**Figure 6:**
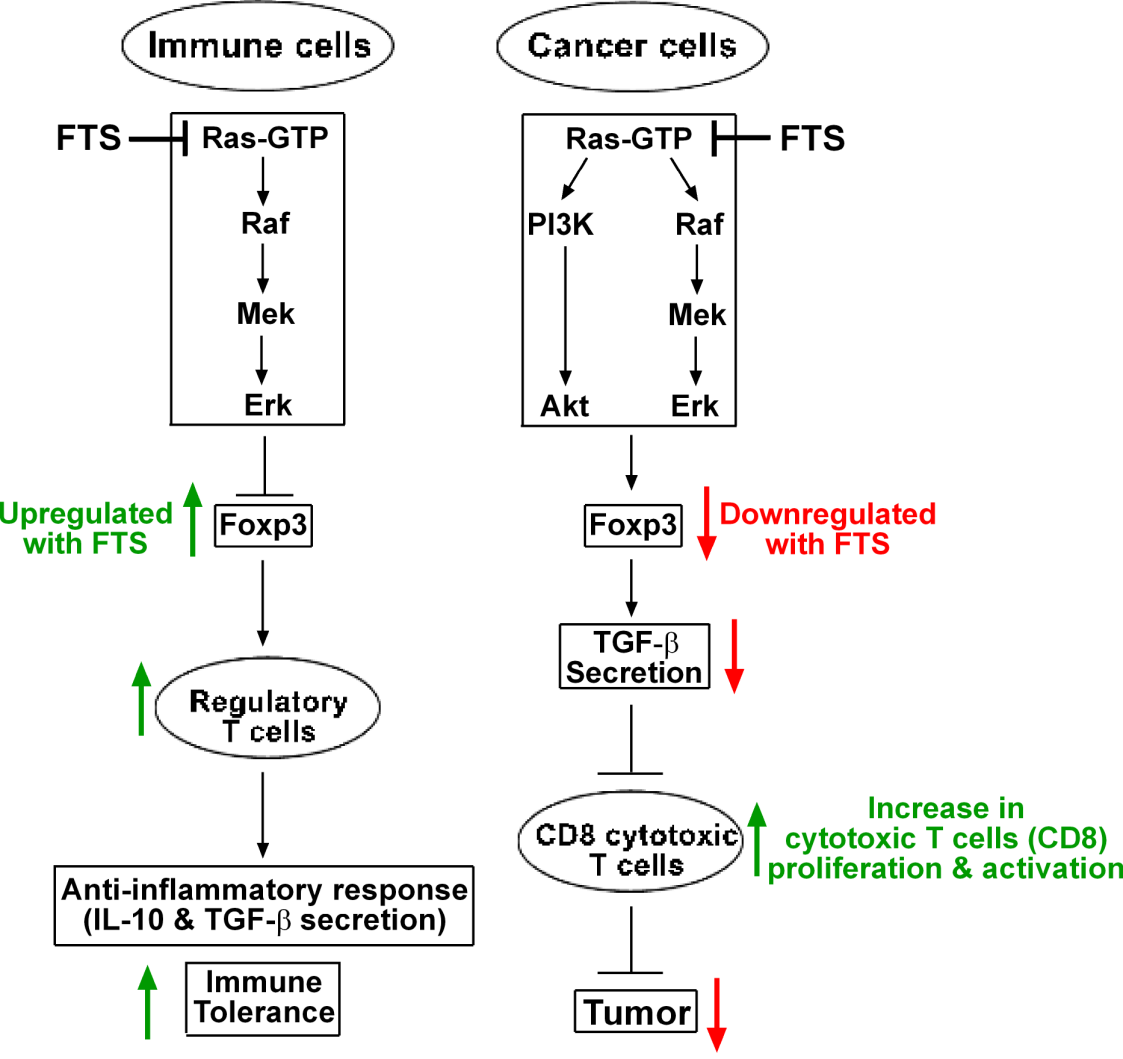
Proposed mechanism explaining the differential effects of Ras inhibition on immune and cancer cells Ras inhibition by FTS acts differently on Foxp3 expression in immune cells and in cancer cells. In immune cells, Ras regulates Foxp3 expression via the MAPK pathway. Its inhibition results in upregulation of Foxp3 expression, thereby augmenting Foxp3^+^ regulatory T cells (Tregs) [3-4]. The upregulated CD25^+^Foxp3^+^ Tregs induce an anti-inflammatory effect by secreting tolerogenic cytokines, such as IL-10 and TGF-β, which attenuate the proliferation of effector T cells and thus help to maintain immune tolerance [42]. In the glioma cancer cells, Ras regulates Foxp3 expression via both the MAPK and the PI3K pathways. Treatment of the cancer cells with FTS results in downregulation of Foxp3, which in turn attenuates expression of the immunosuppressive cytokine TGF-β from glioma cells. A similar effect of Foxp3 on TGF-β expression levels has been demonstrated in melanoma cells [43]. The decrease in TGF-β produces an inflammatory tumor microenvironment, which promotes robust proliferation, migration, and activation of antitumor CTLs. This effect on the CTLs intensifies their antitumor character, resulting in decreased tumor growth.

An interesting question with regard to targeted drug therapy has to do with the way in which nontumor cells and tissues cope with the drug-induced inhibition of major growth and differentiation pathways. The coping mechanisms of the immune system and the microenvironment are of particular interest because of the involvement of these systems in tumor growth [39]. The various drugs inhibit, either directly or indirectly, many signaling molecules in these systems, including MAP kinases, Akt, cyclin-dependent kinases, and small GTPases [40]. These inhibitory activities seem to introduce contradictory situations: on the one hand they might reduce the antitumor activity of many targeted drugs, while on the other hand they support some of their antitumor activity, for example by enhancing the antitumor activity of the immune system. One example of possible contradictory effects is given by FTS, which exhibits potent antitumor activity in mice [23-24] and in humans (http://www.concordiapharma.com), and a strong anti-inflammatory activity in mice [3]. The results of the present study showed, however, that Ras inhibition by FTS in mice provides a favorable antitumor environment both in the immune system and in glioma cells. FTS was found here to have three major effects in immune-competent tumor-bearing mice. First, it exhibited antitumor activity and increased survival of immune-competent mice with intracranial gliomas (Figure 5). Notably, the increased survival of immune-competent C57bl/6 mice relative to nude mice was apparently a function of the presence or absence of an intact immune system, rather than of strain differences. For example, we previously reported that FTS treatment of intracranial U87 gliomas failed to achieve increased survival in nude mice [31]. Those results, like the present findings, indicated that the increase in survival was prevented not by the type of tumor or the strain of mouse, but by an incompetent immune system. Second, FTS was found to induce an increase in the antitumor reactivity of CD8^+^ CTLs by downregulating TGF-β expression in GL261 glioma cells (Figure 4). We attributed this effect, in the present study, to the reduction in Foxp3 resulting from FTS treatment of the tumors in immune-competent mice (see scheme in Figure 6). Interestingly, we have also observed a decrease in Foxp3 in DLD1 colon cancer cells *in vitro* (unpublished data), suggesting that the effect of FTS on cancer cells that express Foxp3 might be more general than previously supposed. Third, in tumor-bearing immune-competent mice, an FTS-induced increase in Tregs was observed in splenocytes (Figure 2). Such an increase has also been reported in other mouse strains besides C57bl/6, including Balb/c, and NOD [3-4, 7-8]. Importantly, although FTS was found here to induce an increase in the Tregs of the splenocytes in C57bl/6 mice, no such effect was observed in the tumors (Figure 3). In addition, depletion of peripheral CD25^+^Foxp3^+^ Tregs in tumor-bearing mice did not enhance the tumor-inhibitory effect of FTS. Evidently, therefore, Foxp3^+^ Tregs do not interfere with the inhibitory effects of FTS. These novel findings demonstrated the antitumor activity of FTS in immune-competent mice. They also demonstrated the negative involvement of Foxp3 in glioma and showed that inhibition of Foxp3 by FTS has a favorable antitumor activity.

Taken together, our results suggest that the impact of FTS-induced Ras inhibition (Figures 1 and 2) on Foxp3 expression in the immune system differs from its impact on Foxp3 expression in cancer cells. In the immune cells it leads to upregulation of Foxp3, whereas in cancer cells it leads to Foxp3 downregulation (see scheme in Figure. 6). The outcome of Ras inhibition in immune cells is an enhanced anti-inflammatory response (increased interleukin-10 and TGF-β) and immune tolerance [7]. Its outcome in GL261 glioma tumor cells, however, is decreased secretion of TGF-β and hence an increase in the proliferation and functional capacity of antitumor CD8^+^ CTLs (see Figure 4 and scheme in Figure 6). All in all, our results highlight the importance of the immune system, and probably also of the tumor microenvironment, in supporting tumor growth. They also support a mechanism by which Ras inhibition in glioma cells changes the tumor microenvironment in a way that reduces resistance of the tumor to the immune system and hence induces significantly increased inhibition of cancer growth.

The importance of these results derives from the fact that they can explain some of the major beneficial effects of Ras inhibitors, as well as of inhibitors that act downstream of Ras. Moreover, these beneficial effects are not restricted to inhibition of tumor growth, but also relate to the microenvironment and the immune system. These are novel findings, which provide, in addition, an experimental framework for examining the impact of other anticancer drugs on cancer and the immune system. Such experiments can be used for the design of drug combinations of anticancer and immunostimulatory drugs.

## MATERIALS AND METHODS

### Cell culture and reagents

The glioma cell line GL261 was a generous gift from the laboratory of Prof. Reuven Stein. The GL261 cell line and splenocytes were cultured in DMEM and RPMI medium, respectively, supplemented with 10% fetal calf serum (Biological Industries, Kibbutz Beit Ha-Emek, Israel), 100 U/ml penicillin and 100 U/ml streptomycin, in a humidified environment with 5% CO_2_ at 37°C. FTS was a gift from Concordia Pharmaceuticals (Fort Lauderdale, FL).

### Western blotting and GTPase pull-down assays

GL261 cells were plated at a density of 1×10^6^ cells per 10-cm plate, grown for 24 hours, and then treated with FTS or 0.1% Me_2_SO_4_ (control). Cells were then lysed in 300 μl of homogenization buffer [41], centrifuged for 10 minutes at 14 000 rpm at 4°C, and the supernatant was collected. Equal amounts of proteins (50–100 μg per lane) were subjected to SDS-PAGE, followed by immunoblotting with the following antibodies (Abs): mouse anti-Pan-Ras (Ab-3) monoclonal Ab (mAb) (Calbiochem, San Diego, CA), anti-CD3 (AbD Serotec, Oxford, UK), anti-Erk (Santa Cruz Biotechnology, CA), anti-P-Erk (Sigma, St. Louis, MO), anti-Akt, anti-P-AKT (Cell Signaling, Danvers, MA), anti-tubulin, and anti-Foxp3 (eBioScience, San Diego, CA). Blots were exposed to the appropriate secondary peroxidase-coupled IgG (Jackson ImmunoResearch Laboratories, West Grove, PA) and subjected to enhanced chemiluminescence (Amersham Pharmacia Biotech, Piscataway, NJ). Protein bands were quantified by densitometry with Image EZQuant-Gel Statistical Analysis Software. Ras-GTP in the lysates was assayed by the glutathione^−^ S-transferase – Ras-binding domain (GST-RBD) pull-down assay, and this was followed by western blotting with anti-Pan-Ras (Ab-3) mAb.

### Determination of Foxp3 expression

RNA was extracted from 1×10^6^ GL261 glioma cells using the RNeasy Mini Kit (Qiagen, Hilden, Germany) according to the manufacturer's protocol. Reverse transcription (RT)-PCR was performed according to the protocol of the Reverse-iT™ 1^st^ Strand Synthesis Kit (ABgene, Epsom, UK). Adenosine phosphoribosyltransferase (APRT) was analyzed using the following primers: APRT forward 5′-GCCTCTTGGCCAGTCACCTGA-3′ and APRT reverse 5′-CCAGGCTCACACACTCCACCA-3′. PCR was carried out with ReddyMix™ PCR Master Mix (ABgene) on a Programmable Thermal Controller (MJ Research, Waltham, MA) at gene-specific conditions. Primer sequences for Foxp3 were: Foxp3 forward 5′- CACCCAGGAAAGACAGAACC -3′ and Foxp3 reverse 5′- GCAAGAGCTCTTGTCCATTGA -3′. The PCR products were subjected to electrophoresis in 2% agarose gel stained with ethidium bromide.

### Cell separation and flow cytometry

Spleens and GL261 tumor tissues were removed from C57bl/6 mice and single-cell suspensions were stained with either phycoerythrin (PE)-labeled anti-CD25 (BC96) mAb or PE-labeled anti-CD8 (LY-2) mAb. Foxp3 was stained according to the manufacturer's instructions (eBioscience). Stained cells were analyzed by FACSCalibur (BD Biosciences, San Diego, CA). CD8^+^ cells were isolated from splenocytes by the BD FACSAria cell sorter (BD Biosciences).

### Cell division tracking and coculturing assays

The isolated CD8^+^ cells were harvested, washed twice in cold PBS, resuspended in PBS at a concentration of 5×10^6^ cells/ml, and stained (for assessment of their proliferation rates) by incubation for 10 minutes at 37°C with the fluorescent dye carboxyfluorescein succinimidyl ester (CFSE; 5-(and 6)-carboxyfluorescein diacetate succinimidyl ester; BD Biosciences; 5 mM, diluted 1/1000). The staining reaction was stopped by two washes with complete medium. The stained CD8^+^ cells (1×10^5^ per well) were then cocultured with GL261 cells (1×10^4^ per well) pretreated with 12.5, 25, or 50 μM FTS in RPMI medium for 96 hours in the presence of 2.5 μg/ml anti-CD28 (37.51) and 5 μg/ml anti-CD3e (145-2C11) monoclonal antibodies (eBioscience). In other experiments, we separated the CFSE-stained CD8^+^ cells from the GL261 cells by using 24-well translucent chambers, pore size 0.4 μm (Greiner Bio-One, Frickenhausen, Germany). FTS-pretreated GL261 cells (1×10^4^ per well) were seeded in the lower chamber in 500 μl RPMI, and the isolated and CFSE-stained CD8^+^ cells were placed on top of the membrane (1×10^4^ per well) in 200 μl RPMI. After 96 hours the supernatant media of the isolated and the nonisolated cultures were collected and proliferation of the CFSE-labeled CD8^+^ T cells in both sets of cultures was analyzed by flow cytometry in three independent experiments. All migration experiments were carried out in duplicate.

### Neutralization of TGF-β

Anti-TGF-β1 mAb (R & D Systems, Ellisville, MO; 1 μg/ml) was used as a blocking antibody to neutralize the activity of TGF-β *in vitro*.

### Assay of TGF-β cytokine

Secretion of TGF-β from GL261 cells treated for 24 hours with 12.5, 25, or 50 μM FTS was measured by ELISA (Linco Research, St. Charles, MO).

### *In-vivo* studies

The study was approved by the Institutional Ethics Committee at the Tel Aviv Sourasky Medical Center, Tel Aviv, Israel (Approval ID: L-11-019 for C57bl/6 mice; L-11-024 for athymic nude mice).

### Subcutaneous implantation of GL261 cells into C57bl/6 mice

C57bl/6 male mice (8-10 weeks old) were housed as described earlier [7]. On day 0, GL261 cells (2×10^6^ cells in 0.1 ml PBS) were implanted s.c. just proximal to the right femur. After 7 days, by which time tumor volumes (measured as previously described [41]) were 0.3–0.5 cm^3^, mice were separated randomly into two groups (40 mice per group). Mice in one group were treated daily with oral FTS, 60 mg/kg, and mice in the other group received vehicle (carboxymethyl cellulose). After 18 days the mice were killed. Tumors were weighed and were then homogenized for western blotting and FACS analysis as described above.

### Intracranial implantation of GL261 glioma cells into C57bl/6 and nude mice

Male C57bl/6 mice (4 months old) and athymic nude mice (8–10 weeks old) were anesthetized by intraperitoneal (i.p.) injection of ketamine (100 mg/kg) and xylazine (20 mg/kg) and then placed in a stereotaxic alignment system (Kopf Instruments, CA). A cut, approximately 1 cm long, was made in the scalp, exposing the skull, and a 2-mm burr hole was drilled 1 mm posterior to the bregma and 1.5 mm lateral to it. A Hamilton 10-μl syringe and a 31-gauge Hamilton needle were used to implant 1×10^5^ GL261 cells in 3 μl DMEM, 3 mm below the surface of the cortex, at a rate of 1 μl/min. To avoid backflow, the needle was left for an additional 1 minute before being gradually removed. The scalp was stitched and the mice were allowed to recover in their cages. Survival rates of the tumor-bearing mice were recorded.

### Magnetic resonance imaging

At 10, 14, and 17 days after intracranial implantation of GL261 cells, tumor progression was assessed by magnetic resonance imaging (MRI) as described earlier [31]. The MRI scans were performed under inhalational isoflurane (1%–2%) anesthesia (Nicholas Piramal (Mumbai, India) in 98% oxygen. Immediately before scanning the mice were injected i.p. with 150 μl of 0.1M Gd-DTPA (Soreq Radiopharmaceuticals, Yavne, Israel). Mice were scanned in a 7T/30 spectrometer (Bruker Biospin, Ettlingen, Germany) using a quadrature head coil and a 400 mT/m gradient system. The MRI protocol included gadolinium (Gd)-DTPA-enhanced T1 weighted imaging (TR=800 and TE=12 ms); field of view 2×2 cm; matrix dimensions 256×128 (reconstructed to 256×256) pixels). Fourteen slices, 0.8 mm thick with no gap, were acquired in the coronal orientation. Final image resolution was 0.078×0.078×1 mm^3^. The tumor area was determined using the Medical Image Analysis version 2.4 in MATLAB (Mathworks, MA).

### Survival analysis

Following their intracranial implantation with GL261 cells, mice were monitored and weighed daily until they died. Survival curves were calculated by the Kaplan-Meier procedure. The log-rank test was used for statistical analysis.

### Downregulation of Foxp3^+^ CD25^+^ cells *in vivo*

Regulatory T cells *in vivo* were decreased by injection of purified anti-CD25 Ab, which blocks the production of CD25^+^ cells (Tregs). Anti-CD25 Ab was contributed by Prof. Jacob George's lab. GL261 cells were implanted s.c. in the flanks of C57bl/6 mice on day 0, as above. On day 4 the tumor-bearing mice were divided into three groups. In two of the groups each mouse was injected i.p. on days 4 and 11, in the first group with 250 μg of purified anti-CD25 Ab and in the second group with 250 μg of functional grade purified IgG1 Ab (eBioscience). From day 5, both groups received FTS treatment. The mice in the third group did not receive antibody and were treated with the vehicle (control) instead of FTS. Tumors were measured on days 6, 10, 17, and 21.

### Statistical analysis

Descriptive analytical data are presented as means ± SEM. Tumor volumes were compared by two-way ANOVA (repeated measures). Survival was assessed by Kaplan-Meier survival analysis followed by a log-rank test. The results of all other experiments were analyzed by unpaired Student's *t*-test and one-way ANOVA. Values of p<0.05 were considered statistically significant.
